# Can neuroimaging predict dementia in Parkinson’s disease?

**DOI:** 10.1093/brain/awy211

**Published:** 2018-08-29

**Authors:** Juliette H Lanskey, Peter McColgan, Anette E Schrag, Julio Acosta-Cabronero, Geraint Rees, Huw R Morris, Rimona S Weil

**Affiliations:** 1 Institute of Neurology, UCL, Queen Square, London, UK; 2 Department of Clinical Neurosciences, University of Cambridge, Cambridge, UK; 3 Huntington’s Disease Centre, UCL, Queen Square, London, UK; 4 Department of Clinical Neurosciences, Royal Free Campus UCL Institute of Neurology, UK; 5 Wellcome Centre for Human Neuroimaging, UCL, Queen Square, London, UK; 6 Institute of Cognitive Neuroscience, UCL, Queen Square, London, UK; 7 Department of Movement Disorders, UCL, Queen Square, London, UK; 8 UCL Dementia Research Centre, Queen Square, London, UK

**Keywords:** Parkinson’s disease, dementia, imaging, MRI

## Abstract

Dementia in Parkinson’s disease affects 50% of patients within 10 years of diagnosis but there is wide variation in severity and timing. Thus, robust neuroimaging prediction of cognitive involvement in Parkinson’s disease is important: (i) to identify at-risk individuals for clinical trials of potential new treatments; (ii) to provide reliable prognostic information for individuals and populations; and (iii) to shed light on the pathophysiological processes underpinning Parkinson’s disease dementia. To date, neuroimaging has not made major contributions to predicting cognitive involvement in Parkinson’s disease. This is perhaps unsurprising considering conventional methods rely on macroscopic measures of topographically distributed neurodegeneration, a relatively late event in Parkinson’s dementia. However, new technologies are now emerging that could provide important insights through detection of other potentially relevant processes. For example, novel MRI approaches can quantify magnetic susceptibility as a surrogate for tissue iron content, and increasingly powerful mathematical approaches can characterize the topology of brain networks at the systems level. Here, we present an up-to-date overview of the growing role of neuroimaging in predicting dementia in Parkinson’s disease. We discuss the most relevant findings to date, and consider the potential of emerging technologies to detect the earliest signs of cognitive involvement in Parkinson’s disease.

## Teaser

Dementia is common in Parkinson’s disease but there is wide variation in severity and timing, and identifying at-risk individuals has proved challenging. Lanskey *et al*. provide an overview of conventional and emerging neuroimaging techniques with the potential to predict dementia in patients with Parkinson’s disease.

## Introduction

Although traditionally considered a movement disorder, Parkinson’s disease is often characterized by cognitive impairment, with dementia affecting 50% of patients within 10 years of diagnosis (Williams-Gray *et al.*, 2013). Subtle cognitive changes are found in some patients with Parkinson’s disease, even early in the disease. Where cognitive deficits do not impact on day-to-day functioning, the term Parkinson’s disease with mild cognitive impairment (PD-MCI) is used (Litvan *et al.*, 2012), with prevalence estimates of 19–42% in newly diagnosed patients (Aarsland *et al.*, 2009, 2010; Yarnall *et al.*, 2014). This is in contrast with Parkinson’s disease dementia (PDD), where cognitive changes are seen in more than one domain and affect daily activities (Emre *et al.*, 2007). Patients vary in the timing and severity of Parkinson’s dementia. Generally, the presence of PD-MCI predicts the development of dementia (Domellöf *et al.*, 2015). However, PD-MCI does not reliably predict transition to dementia since ∼10% of individuals with PD-MCI revert to normal cognition during follow-up (Pedersen *et al.*, 2013; Domellöf *et al.*, 2015).

Robust neuroimaging measures to identify patients with Parkinson’s disease at highest risk for cognitive decline is important for three key reasons: (i) to identify at-risk patients for clinical trials of novel disease-modifying treatments (Athauda *et al.*, 2017); (ii) to provide prognostic information for individuals to plan their future, and enable healthcare providers to plan population health and social needs; and (iii) to uncover mechanistic explanations for underlying disease processes.

Parkinson’s disease is classically associated with Lewy bodies, intracellular inclusions composed of α-synuclein (Spillantini *et al.*, 1997). However, cognitive involvement in Parkinson’s disease is most strongly related to the combination of Lewy bodies with Alzheimer’s pathology, in particular fibrillary amyloid-β and intraneuronal hyperphosphorylated tau tangles (Compta *et al.*, 2011). Evidence is also emerging for a synergistic relationship between α-synuclein and amyloid-β. For example, in a large retrospective study, a strong correlation was shown between extent of neurofibrillary tangles, neuritic plaques and α-synuclein (Irwin *et al.*, 2017). *In vitro* models demonstrate that this relationship is causative, with amyloid-β inducing conformational changes in α-synuclein (Swirski *et al.*, 2014). Intriguingly, certain distribution patterns of pathological inclusion across the cortex at post-mortem are strongly linked with more rapid progression of dementia in life in patients with Parkinson’s disease. Specifically, patients with a high burden of Lewy-related pathology in occipital regions showed more rapid progression to dementia (Toledo *et al.*, 2016).

Axonal involvement appears to be critical in the pathophysiology of Parkinson’s disease and associated cognitive involvement. α-Synuclein accumulation may begin in the axonal compartment (Chung *et al.*, 2009) with dystrophic changes in axons occurring before neuronal loss. Notably, cells that are especially vulnerable in Parkinson’s dementia, including cholinergic cells of the nucleus basalis of Meynert and serotonergic cells of the raphe nucleus share the common morphological phenotype of long axonal projections (Perry *et al.*, 1985; Hale and Lowry, 2011; Wu *et al.*, 2014). Therefore, neuroimaging techniques sensitive to specific pathological accumulation, particularly in occipital regions, and those that detect axonal damage or alterations in neurotransmitter levels are most likely to detect the earliest stages of Parkinson’s dementia.

Until recently, neuroimaging has not had a large role in predicting cognitive involvement in Parkinson’s disease. This is unsurprising, given that conventional methods rely on loss of volume caused by neuronal death, a relatively late event in Parkinson’s dementia (Schulz-Schaeffer, 2010; Hattori *et al.*, 2012). Furthermore, there are few longitudinal neuroimaging studies of the early signs of dementia in Parkinson’s disease. Where these are lacking, cross-sectional studies that detect differences between Parkinson’s disease patients with and without early cognitive involvement can provide insights into the power of these techniques to identify patients likely to progress to Parkinson’s dementia.

A further, important consideration is that cognitive dysfunction in Parkinson’s disease is a heterogeneous entity, especially at the very earliest stages. Two distinct phenotypes are now recognized (Williams-Gray *et al.*, 2009): a fronto-striatal/executive pattern, which is related to dysfunction in dopaminergic fronto-striatal networks; and a posterior cortical/visuospatial phenotype, with a non-dopaminergic substrate, that may involve changes in cholinergic transmission (Klein *et al.*, 2010), or excess cortical protein aggregation, as implicated by association with the *MAPT* genotype (Nombela *et al.*, 2014). In longitudinal population studies, the fronto-striatal phenotype does not always progress to Parkinson’s dementia (Williams-Gray *et al.*, 2013) and therefore imaging techniques sensitive to executive dysfunction may have less value in predicting the earliest stages of Parkinson’s dementia than those sensitive to visuospatial and cholinergic dysfunction.

New emerging technologies show potential for detecting even subtle cognitive involvement in Parkinson’s disease that will need confirmation in longitudinal progression studies. Here we provide an up-to-date overview of the potential role of neuroimaging in predicting dementia in Parkinson’s disease. We consider conventional methods and then examine the sensitivity of emerging technologies to detect the earliest signs of cognitive involvement in Parkinson’s disease.

## Radionuclide imaging

### Metabolic activity

**Figure 1:**
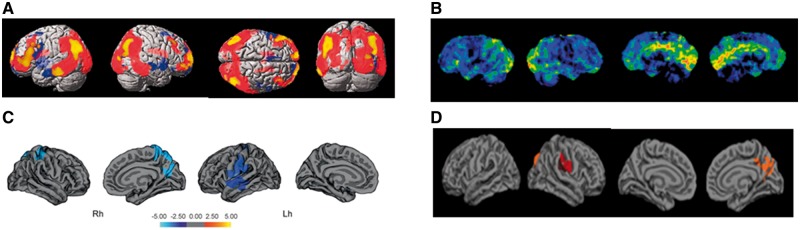
**Cerebral hypometabolism and dementia in Parkinson’s disease and grey matter atrophy in Parkinson’s with cognitive involvement.** (**A**) Regions of cerebral hypometabolism in patients with PDD overlap with regional atrophy. Adapted from González-Redondo *et al.* (2014). (**B**) Statistical maps of baseline ^18^F-FDG-PET data comparing patients with Parkinson’s disease that later develop dementia with controls. Hypometabolism is seen in posterior brain regions particularly in cuneus and precuneus. Image adapted from Bohnen *et al.* (2011). (**C** and **D**) Vertex-wise comparisons of cortical thickness between patients with PD-MCI and Parkinson’s disease without cognitive involvement. Atrophy patterns differ between studies, although atrophy in the precuneus is frequently reported. (**C**) Greatest atrophy seen in left precuneus. Modified with permission from Pereira *et al.* (2014). (**D**) Greatest atrophy seen in precuneus and bilaterally in superior parietal regions. Figure adapted from Segura *et al.* (2014). Lh = left hemisphere; Rh = right hemisphere.

Changes in brain metabolism can be measured using fluorodeoxyglucose (FDG) PET, which is sensitive to glucose uptake and also with single photon emission computed tomography (SPECT), which detects changes in cerebral blood flow. Areas of hypometabolism are seen using FDG-PET in patients with PD-MCI (González-Redondo *et al.*, 2014) with larger areas in PDD (Jokinen *et al.*, 2010), particularly in posterior regions (Garcia-Garcia *et al.*, 2012; González-Redondo *et al.*, 2014; Shoji *et al.*, 2014; Tang *et al.*, 2016) (Fig. 1A). This reduction in temporo-parietal metabolism is also seen in some patients with Parkinson’s disease without dementia, potentially reflecting early posterior cortical involvement in these patients (Hu *et al.*, 2000).

SPECT studies show a similar picture, with reduced cerebral blood flow in patients with PD-MCI (Osaki *et al.*, 2005; Derejko *et al.*, 2006; Nobili *et al.*, 2009), and even greater reductions in PDD (Kawabata *et al.*, 1991; Sawada *et al.*, 1992; Mito *et al.*, 2005; Ma *et al.*, 2008).

Longitudinal studies consistently show involvement of posterior regions in earlier stages of cognitive decline in Parkinson’s disease, with reductions in baseline FDG-PET metabolism in posterior cortical regions in patients that later convert from cognitively-normal to PDD (Bohnen *et al.*, 2011; Tard *et al.*, 2015; Firbank *et al.*, 2017; Baba *et al.*, 2017; Homenko *et al.*, 2017) (Fig. 1B).

Cognitive changes may also be preceded by metabolic increases in other areas. Using a principal components analysis approach to FDG-PET data, frontal as well as parietal metabolic reductions were seen, alongside increases in other areas, including cerebellar vermis and dentate nucleus (Huang *et al.*, 2007; Meles *et al.*, 2015).

### Dopaminergic function

Dopamine projections can be probed *in vivo* with PET or SPECT using markers of dopaminergic terminal integrity and may relate to cognitive involvement in Parkinson’s disease. Decline in cognitive function, particularly executive dysfunction, is associated with loss of caudate uptake on dopamine transporter (DAT) SPECT imaging (Nobili *et al.*, 2010; Arnaldi *et al.*, 2012; Ekman *et al.*, 2012; Lebedev *et al.*, 2014; Siepel *et al.*, 2014; Pellecchia *et al.*, 2015) and with reduced caudate dopaminergic function, as assessed using PET (Brück *et al.*, 2001). Caudate uptake on DAT-SPECT imaging may even predict cognitive decline, especially when combined with other measures including age and CSF (Schrag *et al.*, 2016).

Studies using PET radioligands that bind to dopamine D2 receptors, show reduced D2-receptor availability in the striatum (Monchi *et al.*, 2006; Sawamoto *et al.*, 2008) and orbitofrontal cortex (Ko *et al.*, 2013) of neurologically normal people when performing executive tasks (Monchi *et al.*, 2006; Sawamoto *et al.*, 2008). These reductions are not seen in people with Parkinson’s disease (Sawamoto *et al.*, 2008; Ko *et al.*, 2013), suggesting that the normal striatal and orbitofrontal release of endogenous dopamine in the striatum and orbitofrontal cortex during executive processes is impaired in Parkinson’s disease.

Patients with PD-MCI have reduced availability of D2 receptors in the bilateral insula, compared to patients with Parkinson’s disease and normal cognition, and this availability is positively correlated with executive function (Christopher *et al.*, 2014). Importantly, no between-group differences in cortical thickness are seen in any region, suggesting that loss of D2 receptors in the insula contributes to executive dysfunction in Parkinson’s disease, and that these changes are seen before structural alterations take place.

These studies suggest that executive deficits in Parkinson’s disease are associated with dopaminergic dysfunction in both the striatum and cortex. In comparison to measures of atrophy, PET measures may be more sensitive to cognitive impairment (Christopher *et al.*, 2014). However, as D2-receptor availability is most sensitive to executive dysfunction (Christopher *et al.*, 2014), measures of D2-receptor availability may have less power to identify those individuals that actually progress to Parkinson’s dementia (Kehagia *et al.*, 2012).

### Cholinergic function

Radioligands of cholinergic enzymes allow *in vivo* assessment of cholinesterase activity. Several studies show lower cholinesterase activity in PDD than in Parkinson’s disease without dementia (in patients who were not taking cholinesterase inhibitors) (Kuhl *et al.*, 1996; Hilker *et al.*, 2005; Shimada *et al.*, 2009; Klein *et al.*, 2010), particularly in parietal (Kuhl *et al.*, 1996; Hilker *et al.*, 2005; Shimada *et al.*, 2009; Klein *et al.*, 2010) and occipital regions (Kuhl *et al.*, 1996; Klein *et al.*, 2010). Decreased cortical cholinergic activity is also associated with poorer scores on cognitive testing (Bohnen *et al.*, 2006, 2015; Lorenz *et al.*, 2014). Whether early reductions in cholinesterase activity in parieto-occipital regions is associated with later dementia has not yet been explicitly shown, but in light of converging evidence for the importance of posterior dysfunction and cholinergic deficits as a precursor for Parkinson’s dementia, these are likely to become important future measures.

### Phosphodiesterase 4 expression

^11^C-rolipram PET can measure expression of phosphodiesterase 4, an intracellular enzyme involved in synaptic plasticity and memory. Reduced expression of phosphodiesterase 4 in people with Parkinson’s disease, specifically in caudate, thalamic and frontal regions, correlates with impaired spatial working memory (Niccolini, 2017). Notably, these reductions are observed in the absence of cortical and subcortical atrophy in regional analyses.

### Amyloid-β and tau imaging

α-Synuclein deposition is a key pathological hallmark of Parkinson’s disease and related PDD but there is currently no radioligand that binds to α-synuclein. Other pathological substrates, especially amyloid-β and tau, are strongly linked with PDD (Compta *et al.*, 2011; Irwin *et al.*, 2013) and PET can be used to detect these. In PET studies, Pittsburgh compound B (PiB) binds to amyloid-β. A recent systematic review identified increased amyloid positivity in PDD compared with Parkinson’s disease patients without dementia, where amyloid positivity was defined as those exhibiting Alzheimer’s-range cortical amyloid deposition on PET imaging performed with PiB (Petrou *et al.*, 2015). Across the 11 studies included in the meta-analysis, 21/74 people with PDD were amyloid positive, compared with only 3/60 patients with PD-MCI. Amyloid binding is also negatively correlated with cognition (Gomperts *et al.*, 2013; Akhtar *et al.*, 2017).

In a longitudinal study (Gomperts *et al.*, 2013), increased baseline amyloid burden in Parkinson’s disease without dementia was associated with higher risk of developing cognitive symptoms. However, in the same study, participants with highest baseline PiB-amyloid-β did not develop PDD, and other studies found no difference in intensity (Foster *et al.*, 2010; Gomperts *et al.*, 2012) or pattern (Campbell *et al.*, 2013) of amyloid-β burden between PDD and controls, suggesting that PiB binding alone has low specificity for dementia prediction in Parkinson’s disease.

Tau has been found to co-localize with α-synuclein (Compta *et al.*, 2011) and *MAPT* polymorphism is associated with increased risk of dementia in Parkinson’s disease (Williams-Gray *et al.*, 2013). The radioligand ^18^F-AV-1451 binds strongly to tau (Dani *et al.*, 2016) and a recent cross-sectional study found a correlation between ^18^F-AV-1451 uptake in the precuneus and inferior temporal gyrus with cognitive performance (Gomperts *et al.*, 2016), but this has not yet been confirmed longitudinally.

Unlike PiB or ^18^F-AV-1451, which selectively bind to amyloid-β plaques and neurofibrillary tau tangles, respectively, ^18^F-FDDNP binds to both tau and amyloid-β aggregates. Buongiorno and colleagues (2017) found that binding of ^18^F-FDDNP globally and in lateral temporal regions was higher at baseline in people with Parkinson’s disease who converted to PDD at follow-up than in patients who did not develop dementia and that baseline lateral temporal ^18^F-FDDNP binding correlated with worse performance at later cognitive testing. Given that post-mortem evidence suggests it is the combination of pathological proteins that is most discriminatory for PDD (Compta *et al.*, 2011), it is of particular relevance that the best evidence for pathological protein imaging, in the absence of a specific α-synuclein ligand, is for a ligand sensitive to both amyloid and tau.

### Neuroimaging of neuroinflammation

Increased microglial activation is seen in Parkinson’s disease both within regions with Parkinson’s disease-related pathology and at distant regions (Imamura *et al.*, 2003). Changes in microglial morphology could be in response to local, microenvironment cues (Mrdjen *et al.*, 2018) and may precede the spread of pathology in neurodegeneration (Streit *et al.*, 2009). As microglial activation is linked to increased expression of the mitochondrial translocator protein (TSPO), ligands that bind to TSPO indicate areas of neuroinflammation. A study using a TSPO ligand demonstrated increased cortical microglial activation in patients with Parkinson’s disease (with and without dementia) and identified increased left parietal neuroinflammation in patients with Parkinson’s dementia compared to Parkinson’s patients without dementia (Edison *et al.*, 2013). Importantly, even in Parkinson’s patients without dementia, this measure of microglial activation in temporo-parietal, occipital and frontal areas negatively correlated with cognitive performance. Measures of microglial activity are also sensitive to the earliest stages of dementia with Lewy bodies (Iannaccone *et al.*, 2013), suggesting that imaging measures of neuroinflammation such as these may have a role in predicting Parkinson’s dementia.

## Grey matter measurements

Grey matter atrophy may represent neuronal cell death and has long been associated with cognitive decline in Alzheimer’s disease (Fox and Schott, 2004). This observation led to a large number of neuroimaging studies comparing patients with Parkinson’s disease without cognitive impairment and people with PD-MCI. These showed varying atrophy patterns in frontal (Song *et al.*, 2011; Melzer *et al.*, 2012; Mak *et al.*, 2013; Hanganu *et al.*, 2014; Gao *et al.*, 2017), temporal (Melzer *et al.*, 2012; Hu *et al.*, 2013; Mak *et al.*, 2013; Pagonabarraga *et al.*, 2013; Hanganu *et al.*, 2014; Noh *et al.*, 2014; Gao *et al.*, 2017), occipital (Melzer *et al.*, 2012; Pagonabarraga *et al.*, 2013), parietal (Melzer *et al.*, 2012; Pereira *et al.*, 2014) and insular cortices (Mak *et al.*, 2013; Hanganu *et al.*, 2014), as well as subcortical atrophy (Melzer *et al.*, 2013; Hanganu *et al.*, 2014; Foo *et al.*, 2017; Schneider *et al.*, 2017), including hippocampal (Melzer *et al.*, 2012; Schneider *et al.*, 2017), amygdala (Melzer *et al.*, 2012; Hanganu *et al.*, 2014) and nucleus accumbens volume loss (Hanganu *et al.*, 2014; Foo *et al.*, 2017) (Fig. 1C). Such wide variability may reflect sensitivity differences across grey matter volume estimation methods [voxel-based morphometry (Song *et al.*, 2011; Melzer *et al.*, 2012; Mak *et al.*, 2013), cortical surface-based analyses (Pagonabarraga *et al.*, 2012; Hanganu *et al.*, 2014; Pereira *et al.*, 2014) and region of interest analyses (Choi *et al.*, 2012)], as well as varying definitions of PD-MCI. Studies conducted prior to recent 2012 guidelines for definitions of PD-MCI (Litvan *et al.*, 2012) used differing selection criteria (Song *et al.*, 2011; Pagonabarraga *et al.*, 2013) to studies using the new criteria (Mak *et al.*, 2013; Foo *et al.*, 2017).

Specific cognitive tests also differ between studies (Mak *et al.*, 2013, 2015; Pereira *et al.*, 2014), as well as the weight given to each cognitive domain. Overall, there is lack of evidence to ascertain which cognitive domains are most affected in Parkinson’s disease. Despite methodological heterogeneities, there is some consistency in regions where atrophy correlates with cognitive involvement in Parkinson’s disease. For example, precuneus (Pereira *et al.*, 2014; Segura *et al.*, 2014) and lingual gyrus (Pagonabarraga *et al.*, 2013; Segura *et al.*, 2014) thinning correlates with loss of semantic fluency and visuospatial performance (Fig. 1D), while temporal thinning correlates with memory (Mak *et al.*, 2013; Pagonabarraga *et al.*, 2013; Pereira *et al.*, 2014).

Longitudinal grey matter studies are similarly affected by methodological discrepancies, including different assumptions for the correction of serial data and power, and few studies include large numbers. Despite this, some consistency is emerging. For example, hippocampal thinning is prominent in several studies (Aybek *et al.*, 2009; Weintraub *et al.*, 2012; Morales *et al.*, 2013; Kandiah *et al.*, 2014; Mak *et al.*, 2015; Gasca-Salas *et al.*, 2017; Gee *et al.*, 2017). Another longitudinal study found frontal and cingulate thinning in patients that progressed to PDD, but it was the combination of biomarkers, including CSF, neuropsychological measures and grey matter volume that was most predictive for Parkinson’s dementia (Compta *et al.*, 2013). Ultimately, large prospective studies will be needed to determine the earliest neuroimaging correlates of Parkinson’s dementia. This will be most effectively achieved by large-scale collaboration programmes such as the Parkinson’s Progression Markers Initiative (PPMI, http://www.ppmi-info.org/).

Variations in findings also likely reflect the low sensitivity of grey matter atrophy as a neural correlate of cognitive involvement in Parkinson’s disease. Neuronal cell death, indexed by grey matter atrophy (Rossor *et al.*, 1997), is a relatively late event in the pathogenesis of Parkinson’s dementia (Kurowska *et al.*, 2016). Axonal and synaptic accumulation of pathogenic proteins occurs at an earlier stage, before neuronal loss (Hattori *et al.*, 2013). Therefore, neuroimaging techniques sensitive to changes in axonal microstructure might be better suited to detect the earliest stages of cognitive involvement in Parkinson’s disease.

## White matter changes and diffusion MRI

White matter changes are potentially more sensitive to early processes in Parkinson’s disease as they represent degeneration of axons and myelin damage, which may occur early in the course of the disease (Burke and Malley, 2013). Diffusion-weighted MRI (DWI) can provide *in vivo* information about microstructural integrity both in grey and white matter tissue (Le Bihan *et al.*, 2001). Diffusion tensor imaging (DTI) is a technique that can reliably characterize such restriction by modelling the displacement of water molecules as a rotationally invariant tensor. The diffusion tensor is decomposed into a set of primary components that are then recombined as DTI metrics. These include mean diffusivity, which characterizes the overall molecular displacement, and fractional anisotropy, which indirectly captures the spatial coherence of such displacements, thus reflecting the level of restriction imposed by the parenchymal microstructure. Both metrics generally work on the assumption that water molecules are less coherently restricted as a result of disease processes such as axonal loss.

**Figure 2:**
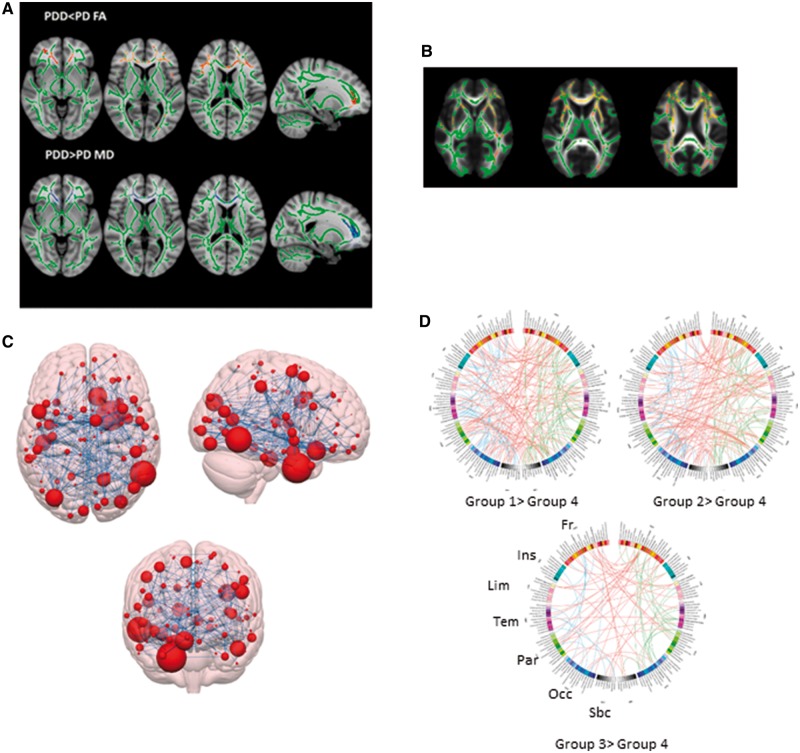
**White matter changes in Parkinson’s with cognitive involvement and changes in brain connectivity associated with cognitive changes in Parkinson’s disease assessed using graph theoretical approaches.** Tract-based spatial statistics results in Parkinson’s patients with differing degrees of cognitive involvement. Voxel-wise group differences are shown in red (decreased fractional anisotropy), overlaid on the white matter skeleton (in green). Comparison of white matter integrity in this way reveals decreased fractional anisotropy and increased mean diffusivity in several major white matter tracts in Parkinson’s patients with cognitive involvement. (**A**) Comparison of PDD and cognitively normal Parkinson’s disease (PD). Adapted from Kamagata *et al.* (2013). (**B**) Associations between mean diffusivity and performance in semantic fluency task. Tract-based spatial statistics map showing areas of increased mean diffusivity (in yellow-red) in the white matter of patients with Parkinson’s disease. A significant association is seen between increased mean diffusivity and lower semantic fluency score. Adapted from Duncan *et al.* (2015). (**C**) Comparisons between controls and PD-MCI using network-based statistics. Schematic representation of the component consisting of 235 edges considered significantly different between the groups. Brain nodes are scaled according to the number of edges in the significant component to which they are connected. Adapted from Abós *et al.* (2017). (**D**) Connectograms comparing patients with Parkinson’s disease divided according to cognitive ability into four groups, where Group 1 is cognitively normal, and Group 4 has dementia. As cognitive impairment worsened, functional connectivity decreased. Between-group differences in functional connectivity especially concerned the ventral, prefrontal, temporal and occipital cortices. Links are coloured by connection type: left intrahemispheric (blue), interhemispheric (red) and right intrahemispheric (green). Brain regions are represented symmetrically. FA = fractional anisotropy; Fr = frontal; Ins = insula; Lim = cingular limbic; Par = parietal; Occ = occipital; Sbc = subcortical; Tem = temporal. Adapted from Lopes *et al.* (2017).

As measured by DTI, white matter alterations in Parkinson’s disease increase as cognition worsens (Kamagata *et al.*, 2013; Melzer *et al.*, 2013; Agosta *et al.*, 2014) (Fig. 2A). Fractional anisotropy is reduced in PDD (relative to controls) in major white matter tracts (Deng *et al.*, 2013; Kamagata *et al.*, 2013). In cross-sectional DTI studies in Parkinson’s disease where white matter and grey matter were analysed concurrently, significant white matter alterations were identified in Parkinson’s patients without dementia where signs of grey matter atrophy were yet unremarkable (Hattori *et al.*, 2012; Agosta *et al.*, 2014; Duncan *et al.*, 2015). Such white matter changes included fractional anisotropy reductions (Hattori *et al.*, 2013; Agosta *et al.*, 2014) and mean diffusivity increases (Duncan *et al.*, 2015) in the inferior and superior longitudinal fasciculi and inferior fronto-occipital fasciculus. In these studies, changes in grey matter were only detectable in patients with dementia (Hattori *et al.*, 2012). This suggests DTI might be more sensitive to changes in white matter microstructure as early signs of cognitive involvement in Parkinson’s disease when compared to measures of atrophy, but further longitudinal studies will be needed to establish the temporal sequence.

When particular cognitive domains are examined in patients with Parkinson’s disease, abnormal tissue diffusivity is seen in specific cortical patterns (measured using fractional anisotropy and mean diffusivity) (Fig 2B). This is found for memory (Carlesimo *et al.*, 2012; Melzer *et al.*, 2013; Zheng *et al.*, 2014), attention (Melzer *et al.*, 2013; Zheng *et al.*, 2014), executive function (Melzer *et al.*, 2013; Theilmann *et al.*, 2013; Zheng *et al.*, 2014), language (Zheng *et al.*, 2014; Duncan *et al.*, 2015), and visuospatial domains (Theilmann *et al.*, 2013). Mean diffusivity of parietal and frontal subcortical tracts is higher in early stage Parkinson’s participants with impaired semantic fluency (Duncan *et al.*, 2015), a measure that has been linked with dementia risk. Moreover, increased mean diffusivity is seen prior to reductions in fractional anisotropy or grey matter volume (Melzer *et al.*, 2013).

Despite the sensitivity of fractional anisotropy and mean diffusivity, these measures are relatively non-specific. Recent advances in diffusion MRI technology allow more accurate quantification of tissue microstructure, in particular for neurite morphology. For example, Neurite Orientation Dispersion and Density Imaging (NODDI) is a technique that can better capture the microstructural complexity of axons and dendrites (Zhang *et al.*, 2012). It has been suggested that this technique might be more sensitive to cortical and subcortical changes in Parkinson’s disease than traditional voxel-based morphometry or surface-based cortical thickness estimations (Kamagata *et al.*, 2017), but it has not been specifically used to study patients with Parkinson’s dementia. More recently, a bi-tensor model has been applied to MRI diffusion data that separates the diffusion properties of water within brain tissue from water in extracellular space. In this way, free water within brain structures can be estimated. This technique may detect higher levels of free water in the posterior substantia nigra for patients with worse cognitive scores (Planetta *et al.*, 2016) and higher levels of free water predicted change in cognitive score after one-year follow-up (Ofori *et al.*, 2015).

## Event-related functional MRI

Event-related functional MRI can be a sensitive indicator of presymptomatic cognitive dysfunction as it may reveal changes in blood oxygen level-dependent signals, reflecting altered patterns of neuronal activity, before gross structural changes are seen. Several groups have shown reduced activation in fronto-parietal regions including ventrolateral prefrontal cortex (VLPFC), dorsolateral prefrontal cortex (DLPFC) and caudate in Parkinson’s patients compared with controls, during executive tasks (Monchi, 2004; Monchi *et al.*, 2007; Baglio *et al.*, 2011; Gawrys *et al.*, 2014; Gerrits *et al.*, 2015; Trujillo *et al.*, 2015). However, these studies did not distinguish between different levels of cognitive function amongst patients with Parkinson’s disease.

Lewis *et al.* (2003) used performance in an executive task (the Tower of London task) to differentiate cognitive function within patients with Parkinson’s disease. They found that patients with poorer executive performance showed reduced activity in VLPFC, DLPFC and caudate whilst performing a memory task. Interestingly, during executive task performance, prefrontal cortex and caudate activities show a non-linear relationship between disease severity and activation, which relates to dopamine treatment (Rowe *et al.*, 2008). Nagano-Saito (2014) showed reduced activity in VLPFC, DLPFC and caudate whilst planning set-shifting, in PD-MCI compared with Parkinson’s disease and no cognitive involvement. During a memory task, PD-MCI patients showed reduced activity in anterior cingulate and caudate compared with Parkinson’s disease patients without cognitive involvement. Importantly, they also showed that dopamine transporter binding correlated with blood oxygen level-dependent activity in caudate, suggesting a neurochemical substrate for these changes. However, working memory and executive deficits—caused by dysfunction in fronto-striatal networks—are not necessarily a precursor of dementia in Parkinson’s disease. Therefore, these studies, whilst sensitive to executive dysfunction, may not be the most useful in identifying the earliest networks linked to Parkinson’s dementia. It is instructive that one group (Baglio *et al.*, 2011) showed additional reduced activation in occipital regions in Parkinson’s disease, even during an executive task, suggesting that changes in occipital lobe activity may reflect preclinical cognitive dysfunction.

Despite evidence for the importance of visuospatial deficits as early precursors of cognitive dysfunction, there are few event-related functional MRI studies examining changes in brain activity related to visuo-spatial dysfunction in Parkinson’s disease, and those that do are focused on visual hallucinations. For example, Meppelink *et al.* (2009) showed reduced blood oxygen level-dependent activity in lateral occipital cortex in patients with Parkinson’s disease in the seconds before an image was recognized, but how this relates to cognitive dysfunction was not explored. Nemcova *et al.* (2017) showed reduced activation during an object-viewing task in the superior parietal lobe in PD-MCI compared with Parkinson’s disease patients with normal cognition. Interestingly, the two groups did not differ in task accuracy, suggesting that altered activation in this brain region may precede loss of visuo-spatial function.

## Combined functional neuroimaging with genotyping

Functional MRI of patients stratified for genes known to be implicated in Parkinson’s dementia provides important insights into potential underlying substrates of cognitive heterogeneity in Parkinson’s disease. For example, Winder-Rhodes *et al.* (2015) used event-related functional MRI to measure brain activations during a memory test involving picture encoding. They related common microtubule associated protein tau (*MAPT*) haplotypes to memory function and showed that hippocampal activation was lower in *MAPT* H1 homozygotes than in H2 carriers. Nombela and co-workers (2014) examined the effects of common variants implicated in Parkinson’s disease cognition on neural activity during tasks specific to three separate cognitive domains: visuospatial performance, executive functions and memory. Their visuospatial mental rotation task revealed reduced parietal activation and impaired visuospatial performance particularly for *MAPT* H1 homozygotes. Intriguingly, they found a relationship between *COMT* met/met homozygotes and executive function in prefrontal cortex and caudate that was strongly related to dopamine dose. They found no relationship between *COMT* or *MAPT* and neural activity during the memory task, but noted an association between *APOE4* allele carrier status and temporo-parietal activation during this task. Two earlier studies by the same group showed underactivation in a fronto-parietal network in *COMT* met/met homozygotes during attentional control and planning tasks (Williams-Gray *et al.*, 2007, 2008). These combined neuroimaging and genotyping studies provide evidence for neurochemical and neuropathological underpinnings for the two distinct patterns of cognitive dysfunction in Parkinson’s disease. *COMT*-associated changes in fronto-parietal regions point to dopaminergic networks and *MAPT*-linked visuospatial and hippocampal deficits implicate tau involvement in Parkinson’s patients with prominent posterior cortical changes.

## Resting state functional MRI

Resting state functional MRI (rs-fMRI) measures blood oxygen level-dependent signal fluctuations when participants are at rest. One method to identify signals obtained in such circumstances related to particular networks is to use independent component analysis (ICA), a technique that mathematically separates rs-fMRI data into independent components. ICA analysis of rs-fMRI data in brain networks may be sensitive to differences in cognitive performance. The most studied is the default mode network (DMN). DMN connectivity within medial and lateral occipito-parietal regions was increased in patients with PD-MCI compared to patients without cognitive impairment (Baggio *et al.*, 2015). This is at odds with most other studies of DMN connectivity in Parkinson’s dementia, which generally show reduced functional connectivity (Tessitore *et al.*, 2012; Yao *et al.*, 2014; Karunanayaka *et al.*, 2016), and may be accounted for by differences in levodopa, which can alter DMN connectivity (Krajcovicova *et al.*, 2012) or may reflect compensatory changes. Interestingly, increased connectivity was correlated with poorer visuo-perceptual scores (Baggio *et al.*, 2015), consistent with importance of loss of visuo-perceptual function early in Parkinson’s-associated dementia.

## Quantitative susceptibility MRI

Selective neuronal vulnerability that predisposes to cognitive involvement in Parkinson’s disease may relate to oxidative stress due in part to excessive brain iron deposition (Dias *et al.*, 2014). Iron has the capacity to generate free-radical species that may precipitate the production of pathological α-synuclein (Li *et al.*, 2011). Susceptibility-weighted imaging (SWI) and quantitative susceptibility mapping (QSM) are relatively new MRI techniques that show promise as proxies for regional cellular vulnerability due to iron accumulation. Both SWI (Schwarz *et al.*, 2014) and QSM (Murakami *et al.*, 2015; Langkammer *et al.*, 2016; Du *et al.*, 2017) have shown sensitivity levels in Parkinson’s disease greater than ever before with MRI. Indeed, a recent whole-brain QSM study revealed widespread cross-sectional changes across brainstem and cortex in a Parkinson’s disease cohort without dementia for which no abnormalities were detected using structural or DTI measures (Acosta-Cabronero *et al.*, 2016). In addition, a longitudinal SWI study found changes on follow-up consistent with increased iron content in several basal ganglia structures that were partly associated with cognitive decline (Rossi *et al.*, 2014). Taken together, these studies suggest that SWI and QSM can detect processes that are highly relevant to the cognitive aspects of Parkinson’s disease.

## Connectomics and graph theory

Connectomics is an emerging field, where neuroimaging data are used to generate brain networks. Functional and structural brain networks can be constructed using rs-fMRI and diffusion tractography, respectively. The brain is divided into distinct regions based on structural or functional information. Each brain region represents a node that may be connected to other nodes in the brain network. In structural brain networks these connections represent anatomical white matter connections, whereas in functional brain networks, connections represent temporal correlations from functional MRI time series (Fornito and Bullmore, 2015).

The topological characteristics of brain networks are described using a mathematical approach known as graph theory (Rubinov and Sporns, 2010). This quantifies relationships across the brain network. It includes measures of network segregation such as the clustering coefficient, which represents the fraction of a node’s neighbours that are connected to each other (Glossary).

Glossary of graph theory metrics

**Area under the curve:** Graph theory metrics are calculated across a range of sparsity thresholds and the area under the curve is calculated in order to avoid choosing an arbitrary threshold value.

**Betweenness centrality:** The fraction of all shortest paths in the network that pass through a given node.

**Clustering coefficient:** Fraction of a node’s neighbours that are connected to each other.

**Degree:** Represents the number of binary connections a brain region has.

**Edge:** Connections between brain regions defined using structural or functional brain imaging.

**Eigen vector centrality:** A measure that assigns increasing importance to a node if both they and their neighbours are highly connected to hub regions.

**Global efficiency (GE):** Inverse of shortest average path length.

**Modularity:** Subdivision of the network such that a module is defined as a group of regions that are highly connected to each other with minimal connections outside the group.

**Motif:** Patterns of interconnections occurring in a brain network at a significantly higher frequency than those occurring in randomized networks.

**Network-based statistics (NBS)**: This is a statistical method that controls the family-wise error rate when mass univariate tests of all connections in a connectome are undertaken.

**Node:** A brain region usually defined using a brain atlas

**Path length (PL):** Minimum number of connections that must be traversed to go from one node to another.

**Small worldness:** The optimal balance between local clustering (clustering coefficient) and shortest path length.

**Strength:** A weighted version of degree that represents the sum of connection weights for a given brain region.

**Thresholding:** Applying a threshold below which connections are removed from the connectivity matrix. This is used to remove spurious connections and create sparsity in the matrix. Many methods are available; however, there is currently no consensus with respect to the optimal approach.

**Weighting:** Brain networks can be binary, where connections or either absent or present, or weighted based on diffusion metrics, such as fractional anisotropy, or the magnitude of temporal correlation between time series in the case of rs-fMRI.

### Functional connectomics

Rs-fMRI is commonly used in Parkinson’s disease connectomics and cognition. However, network construction methodologies vary, making comparisons across studies difficult (Table 1). One study revealed higher clustering coefficients and modularity in PD-MCI than in patients without cognitive involvement (Baggio *et al.*, 2014), suggesting increased segregation of the functional connectome. Consistent with this, higher clustering coefficients were associated with impaired performance on visuo-spatial tasks.

**Table 1:** Summary of connectome studies investigating connectivity and cognition in Parkinson’s disease

Author	Modality	Patient groups (*n*)	Connectome construction	Analysis	Global changes	Regional changes
Abós *et al.*, 2017	fMRI	HC (38), PD-non-MCI (43), PD-MCI (27)	Weighted, no threshold	NBS (PD-MCI versus HC)	–	Occipito-temporal, occipito-frontal
Baggio *et al.*, 2014	fMRI	HC (36), PD-non-MCI (43), PD-MCI (23)	Weighted, density threshold	GT (ANOVA and cognitive score correlations)	↑ Mod/↑ CC	Tempo-parietal, fronto-temporal
Galantucci *et al.*, 2017	DWI	HC (41), PD-non-MCI (54), PD-MCI (54)	Weighted, no threshold	GT/NBS (*PD-MCI versus HC, ^+^PD-non-MCI versus HC, ^Δ^PD-MCI versus PD-non-MCI)	↑ CC/↓ GE	*^+Δ^Frontal, *^+Δ^temporal, *^+Δ^parietal, *^+^occipital, *^+Δ^cingulate, *^+Δ^BG
Pereira *et al.*, 2015	MRI	HC (56), PD-non-MCI (90), PD-MCI (33)	Weighted, density threshold	GT (*PD-MCI versus HC, ^+^PD-MCI versus PD-non-MCI)	↓ GE/↑ PL	*^+^Superior frontal, *superior parietal, ^+^inferior parietal
Lopes *et al.*, 2017	fMRI	PD (156), cognitive subgroups	Weighted, AUC	GT/NBS (ANOVA across cognitive groups)	↓ CC/↑ GE	Interhemispheric
Luo *et al.*, 2015	fMRI	HC (47), PD (47)	Binary, AUC	GT (cognitive score correlations)	↓ CC/↓ LE	Meso-cortical - visuospatial
Lebedev *et al.*, 2014	fMRI	PD (30)	Weighted, no threshold	GT (cognitive score correlations)	–	Frontal, parietal, limbic, BG
Koshimori *et al.*, 2016	fMRI	HC (25), PD (45)	Binary, density threshold	GT (cognitive score correlations)	–	NS

Loss of connections between specialized brain regions (e.g. occipito-temporal) and between cerebral hemispheres (inter-hemispheric) are commonly associated with PD-MCI. Conflicting results with respect to global graph theory metrics (i.e. CC and GE) are likely due to methodological differences in connectome construction.

AUC = area under the curve; BG = basal ganglia; CC = clustering coefficient; fMRI = functional MRI; GE = global efficiency; GT = graph theory; HC = healthy controls; LE = local efficiency; MNI = Montreal Neurological Institute; Mod = modularity; NBS = network-based statistics; NS = not significant; PD-non-MCI = Parkinson’s disease without mild cognitive impairment or dementia; PL = path length.

^*+^^Δ^Symbols in ‘Analysis’ column indicate which comparisons are referred to in the ‘Regional changes’ column.

In contrast, markers of increased segregation: higher clustering coefficient and reduced global efficiency were associated with a ‘better’ cognitive phenotype in another study (Lopes *et al.*, 2017). Increased segregation may relate to increased connections within specialized brain modules or loss of connections between brain modules, and depending on the balance of these, could be associated with either improved or worsened cognition. Conflicting results may also arise due to methodological differences in calculating graph theory metrics.

A machine learning approach was recently used to classify PD-MCI and Parkinson’s disease without cognitive involvement based on functional connectomes (Abós *et al.*, 2017) (Fig. 2C). Connections used in the classification procedure correlated with executive and visuospatial scores. A network-based statistics (NBS) analysis revealed reduced connectivity in occipital-temporal and occipital-frontal connections in PD-MCI compared to controls. This suggests that inter-regional connections, particularly those involving the occipital lobe, are associated with cognitive impairment in Parkinson’s disease. Correlations between connectivity of visuospatial modules and cognitive performance are even seen in early, drug-naïve Parkinson’s disease (Luo *et al.*, 2015).

Another group investigated differences in functional connectomes across cognitive subgroups in Parkinson’s disease (Lopes *et al.*, 2017) (Fig. 2D). A cluster analysis was performed on a neuropsychological battery splitting patients into five phenotypes, from cognitively intact to severe deficits across cognitive domains. NBS analysis revealed associations between cognitive phenotype and connections involving frontal, temporal, occipital and basal ganglia regions. Separating connections into interhemispheric and intrahemispheric subtypes showed that interhemispheric connections differed between phenotype extremes. This suggests that loss of connections between hemispheres may impact on cognition in Parkinson’s disease [similar associations are seen in Huntington’s disease (McColgan *et al.*, 2017)].

### Structural connectomics

Global network changes in PD-MCI are also seen using structural connectomics (Galantucci *et al.*, 2017), where higher clustering coefficient and reduced global efficiency were found in PD-MCI compared to Parkinson’s patients without cognitive involvement. This is consistent with the model that PD-MCI is associated with increased network segregation and reduced integration and that loss of connections between functional brain modules impairs cognitive functions that require inter-module cooperation (Lopes *et al.*, 2017).

### Connectomics and regional gene expression

Brain structure and function at the macrostructural level can now be linked to gene expression at a cellular level using atlases of gene expression microarray data (Hawrylycz *et al.*, 2012, 2015). In Parkinson’s disease, regions with the largest reductions in connection strength show highest regional expression of *MAPT* (Rittman *et al.*, 2016). In health, brain regions with long-range connections are enriched for genes involved in oxidative metabolism and mitochondrial function (Vértes *et al.*, 2016). This is in keeping with the observation that genetic mutations associated with Parkinson’s disease frequently affect these pathways (Helley *et al.*, 2017).

Taken together, connectomic studies of cognitive impairment in Parkinson’s disease suggest loss of network segregation and integration, affecting hub regions specifically, with prominent loss of connections between hemispheres and specialized functional modules, particularly in posterior brain regions. However, integrating findings across studies is difficult because of methodological differences and the use of unselected Parkinson’s disease cohorts where cognitive phenotypes are ill-defined. These studies highlight the potential for connectomics to identify vulnerable networks and connections involved in PDD. As graph-theoretical methodologies are refined and applied consistently across groups, or within large-scale collaborative studies, they will have increasing importance for early neuroimaging detection of cognitive involvement in Parkinson’s disease.

## Neuroimaging and multimodal predictors of Parkinson’s dementia

Recently, large-scale collaborations have enabled researchers to combine clinical, demographic, biological and genetic factors to determine predictors for Parkinson’s dementia. Liu *et al.* (2017) developed a specific and sensitive algorithm for global cognitive impairment in a large dataset comprising over 3000 patients across nine cohorts. Their algorithm, which includes factors such as age at onset, gender, depression and motor scores, as well as baseline Mini-Mental State Examination, has the advantage of being cheap and non-invasive. It therefore has potential for widespread uptake for disease stratification. However, mean time from disease onset was >6 years. Neuroimaging has the potential to identify at-risk patients far earlier along the disease course and even before they exhibit reduced performance on standard cognitive tests. Other groups have combined clinical and neuroimaging measures in large-scale collaborations, capitalizing on the PPMI programme. Schrag and colleagues (2016) reported good accuracy (area under the curve 0.80) for an algorithm to predict cognitive impairment at 2 years combining clinical measures (excluding baseline cognitive score), CSF parameters and DAT SPECT imaging results at the time of diagnosis. Another recent study (Fereshtehnejad *et al.*, 2017) used clinical, CSF and neuroimaging markers in a data-driven approach to stratify patients with Parkinson’s disease into distinct clusters based on progression and showed a separate rapidly progressing diffuse malignant subtype. The neuroimaging measures in that study were deformation-based morphometry, a method of identifying disease-specific atrophy patterns, and SPECT imaging using a DAT tracer. Although comparisons between clinical subtypes did not survive multiple comparison testing, partly because neuroimaging was available in only a subset, the principle of applying these metrics in combination with other clinical measures shows important potential for defining Parkinson’s subtypes. DAT SPECT imaging, structural MRI and DTI were also used in combination with other clinical and biological modalities by Caspell-Garcia (2017) to examine predictors of cognitive impairment. They showed that predictors of cognitive impairment were linked with dopamine deficiency (*COMT* and *BDNF* polymorphisms, and ipsilateral DAT availability). Although whether identified patients develop persistent dementia over time will need to be determined with longer follow-up. Decreased volume in widespread brain regions also predicted cognitive impairment, particularly in frontal, parietal, temporal and occipital regions. So far, these early findings in large longitudinal cohorts are relatively non-specific. They suggest that the right neuroimaging techniques, in combination with other multimodal measures, may have a role to predict the earliest stages of cognitive involvement, as well providing important insights into underlying pathophysiological mechanisms of Parkinson’s dementia.

## Conclusion

In its current state, neuroimaging is still not able to accurately predict dementia in patients affected by Parkinson’s disease. However, new techniques sensitive to tissue microstructure/biochemical alterations that reflect the very earliest stages of cognitive involvement are now becoming available. The most predictive technologies are likely to be sensitive to axonal damage, show specificity for underlying neuropathological substrates, such as ligands that bind to tau and amyloid, and may involve multimodal approaches. They will need to be specifically tested longitudinally in large-scale studies of patients with Parkinson’s disease to assess their role in early detection of cognitive involvement and ultimately in predicting Parkinson’s dementia. The current move towards large-scale, international collaborative imaging initiatives, especially in combination with other clinical and biological modalities is an essential step towards better-powered, longitudinal imaging studies to provide insights into the biology underlying dementia in Parkinson’s disease and ultimately pave the way for therapeutic interventions aimed at slowing the development of dementia in Parkinson’s disease.

## Funding

R.S.W. is supported by a Clinical Research Career Development Fellowship from the Wellcome Trust (201567/Z/16/Z) and has received funding from UCL, the Academy of Medical Sciences and the National Institute for Health Research University College London Hospitals Biomedical Research Centre (BRC302/NS/RW/101410). GR is supported by the Wellcome Trust. J.L. has no funding sources to declare. J.A.C.: The Wellcome Centre for Human Neuroimaging is supported by core funding from the Wellcome (203147/Z/16/Z). H.R.M. is supported by Parkinson’s UK, the NIHR UCLH BRC, Wellcome Trust, Guarantors of Brain, the Drake Foundation, Cure Parkinson’s Trust, S Koe Research Fellowship, CBD Solutions, the PSP Association and the MRC. A.E.S. is supported by grants from Parkinson’s UK, ESRC, NIHR, GE Healthcare (23/5/13 PO2580367614), EU Commission and the National Institute for Health Research University College London Hospitals Biomedical Research Centre.

## Competing interests

A.E.S. reports personal fees from Medtronic and AstraZeneca. H.R.M. reports personal fees from Teva, AbbVie, Boehringer Ingelheim, and GSK. R.S.W. reports personal fees from GE Healthcare

## Abbreviations

DAT: dopamine transporter
DTI: diffusion tensor imaging
FDG: fluorodeoxyglucose
MCI: mild cognitive impairment
PDD: Parkinson’s disease dementia
PD-MCI: Parkinson’s disease with mild cognitive impairment
PiB: Pittsburgh compound B
SPECT: single photon emission computed tomography
